# Analysis of gut microbiota in chinese donkey in different regions using metagenomic sequencing

**DOI:** 10.1186/s12864-023-09575-z

**Published:** 2023-09-05

**Authors:** Rong Guo, Wei Zhang, Wei Shen, Guoliang Zhang, Taifeng Xie, Ling Li, Jiacuo Jinmei, Yiduan Liu, Fanyong Kong, Baozhu Guo, Benke Li, Yujiang Sun, Shuqin Liu

**Affiliations:** 1 College of Animal Science and Technology, Qingdao Agricultural University, Qingdao, Shandong China; 2 Gene Bank of Equine Genetic Resources, Qingdao, Shandong China; 3 Vocational College of Dongying, Dongying, Shandong China; 4 College of Life Sciences, Qingdao Agricultural University, Qingdao, Shandong China; 5 Tibet Autonomous Region Animal husbandry Station, Tibet, China; 6 Yunnan Provincial Animal Husbandry Station, Yunnan, China; 7 Honghe state animal husbandry technology extension station, Honghe, Yunnan China; 8 Zhangjiakou City animal husbandry technology extension station, Zhangjiakou, Hebei China; 9 Binzhou City Agricultural Technology Extension Center, Binzhou, Shandong China

**Keywords:** Chinese donkey, Gut microbes, Metagenome

## Abstract

**Background:**

Gut microbiota plays a significant role in host survival, health, and diseases; however, compared to other livestock, research on the gut microbiome of donkeys is limited.

**Results:**

In this study, a total of 30 donkey samples of rectal contents from six regions, including Shigatse, Changdu, Yunnan, Xinjiang, Qinghai, and Dezhou, were collected for metagenomic sequencing. The results of the species annotation revealed that the dominant phyla were Firmicutes and Bacteroidetes, and the dominant genera were *Bacteroides*, *unclassified_o_Clostridiales* (short for *Clostridiales*) and *unclassified_f_Lachnospiraceae* (short for *Lachnospiraceae*). The dominant phyla, genera and key discriminators were Bacteroidetes, *Clostridiales* and Bacteroidetes in Tibet donkeys (Shigatse); Firmicutes, *Clostridiales* and *Clostridiales* in Tibet donkeys (Changdu); Firmicutes, *Fibrobacter* and Tenericutes in Qinghai donkeys; Firmicutes, *Clostridiales* and Negativicutes in Yunnan donkeys; Firmicutes, *Fibrobacter* and Fibrobacteres in Xinjiang donkeys; Firmicutes, *Clostridiales* and Firmicutes in Dezhou donkeys. In the functional annotation, it was mainly enriched in the glycolysis and gluconeogenesis of carbohydrate metabolism, and the abundance was the highest in Dezhou donkeys. These results combined with altitude correlation analysis demonstrated that donkeys in the Dezhou region exhibited strong glucose-conversion ability, those in the Shigatse region exhibited strong glucose metabolism and utilization ability, those in the Changdu region exhibited a strong microbial metabolic function, and those in the Xinjiang region exhibited the strongest ability to decompose cellulose and hemicellulose.

**Conclusion:**

According to published literature, this is the first study to construct a dataset with multi-regional donkey breeds. Our study revealed the differences in the composition and function of gut microbes in donkeys from different geographic regions and environmental settings and is valuable for donkey gut microbiome research.

## Introduction

Gut microflora is closely associated with host health and has achieved significant attention owing to its crucial role in the body’s metabolism, immunity, and physiological functions [1–3]. All equines are members of the family of herbivorous mammals and possess a specific hindgut (cecum and colon) microbiota, which can utilize forage for optimal nutrition. These microorganisms contribute to a significant portion of a horse’s daily energy needs by fermenting plant material into short-chain fatty acids such as acetate, propionate, and butyrate [4]. The hindgut of a donkey shares some similarities with ruminants and monogastrics, like ruminants, the contents of the cecum and colon are combined, but unlike ruminants, donkeys do not regurgitate digestion for further chewing and do not have a complex forestomach to help digest plant carbohydrates and proteins [5]. Donkeys are highly dependent on their gut microbiota for nutrition, as these provide glycoside hydrolases and polysaccharide lyases to digest complex polysaccharides that make up the bulk of their carbohydrate intake, and gut microbes convert “difficult” substrates such as cellulose, hemicellulose, and pectin into useful nutrients such as short-chain fatty acids [6].

Li et al. [7] used metagenomics to sequence horse intestinal samples and observed that a high abundance and diversity of antibiotic resistance genes (ARG) were identified in the myelin-associated glycoprotein, which demonstrated the horse gut as a reservoir of ARGs. Although donkeys and horses are closely related, their gut microbiomes markedly differ in composition and structure. On comparing the composition and function of the microbiomes of Przewalski’s proto antelope and Asiatic wild ass using a metagenomic shotgun sequencing method, Tang et al. [8] discovered that homologous wild horses have different microbial compositions while having stable microbial functional cores, which may help them survive in challenging habitats. Li et al. [9] observed a complex relationship between the gut microbiome and gene expression in their study on the hindgut microbial community of donkeys, especially in the immune system, and that the peroxisome proliferator-activated receptor pathway is mainly enriched in the cecum. The hindgut of donkeys shares some similarities with that of ruminants and monogastric animals, such as the combined contents of the cecum and colon. However, contrasting with ruminants, donkeys do not regurgitate the digesta for further chewing and have no complex foregut to aid the digestion of plant carbohydrates and proteins. Donkeys are highly dependent on their gut microbiota for nutrition since these microbiota secrete glycoside hydrolases (GH) and polysaccharide lyases to digest the complex polysaccharides, which account for the majority of their carbohydrate intake. “Difficult” substrates, such as hemicellulose and pectin, are converted into essential nutrients such as short-chain fatty acids [10]. The gut microbiota constitute a complex ecosystem that is involved not only in digestive function [11] but also in immune function [12, 13], gut-brain connectivity and behavior [14], diabetes, and obesity [4, 15]. Although in some cases it can also become an enteric pathogen [16]. China has various ecology types including the plateau continental climate, the temperate monsoon climate of the North China Plain, and the subtropical plateau monsoon climate, which comprises abundant donkey species resources. Particularly, Qinghai-Tibet Plateau is famous for its high-altitude, low-temperature, and hypoxic conditions. Moreover, the emergence of metagenomics has provided microbial ecology a technical boost, and human society has benefited from the progressive revelation of the roles of gut microbes.

In this study, metagenomic sequencing was used to conduct species annotation and gene annotation research on the donkey rectal microbiome in six regions and provide a data platform for the subsequent mining of functional genes. The importance of this paper lies in studying the differences in the composition and function of donkey gut microorganisms under different regional and environmental conditions, understanding the influence of intestinal flora on the metabolic homeostasis of donkeys, and providing important theoretical guidance for improving the microecological environment of donkey gastrointestinal tract and preventing and controlling the occurrence of diseases.

## Materials and methods

### Sample collection, DNA extraction and metagenomic sequencing

**Fig. 1:**
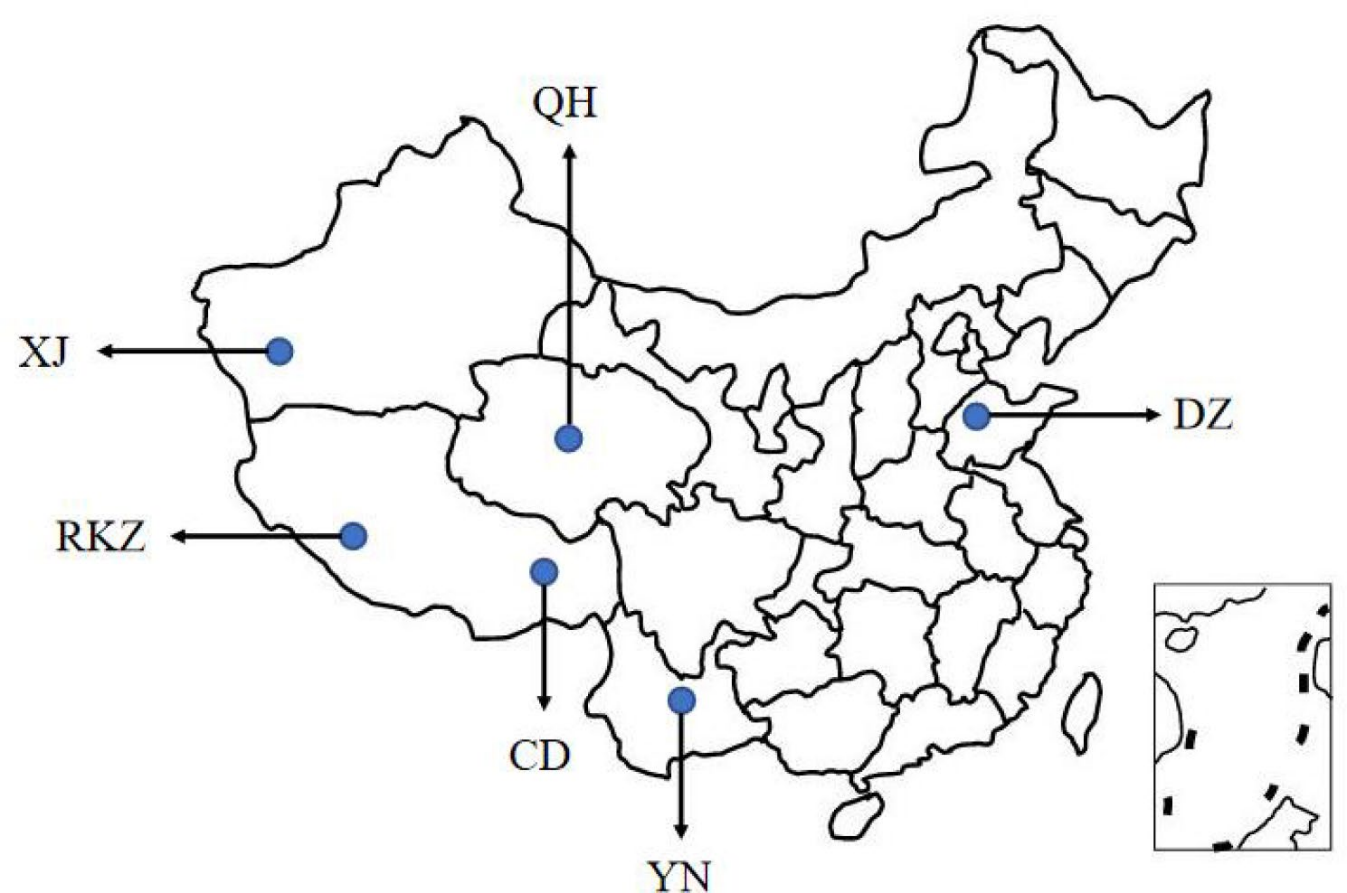
Geographical distribution of donkeys in six regions. Shigatse (RKZ), Changdu (CD), Qinghai (QH), Yunnan (YN), Xinjiang (XJ), and Dezhou (DZ).

We collected five rectal contents of donkeys from each region, with a total of 20 samples from four areas, including Shigatse located in Tibet (RKZ, 5,000 m above sea level), Changdu located in Tibet (CD, 3,500 m above sea level), Yunnan (YN, 2,000 m above sea level), and Xinjiang (XJ, 1 200 m above sea level) (Fig. 1). All sampling subjects were adult healthy donkeys aged 5–10 years old with medium condition, feeding with local feed. All collected samples were guaranteed to be consistent with other environmental elements like temperature, humidity, etc. A rectal sampling method was employed. Long disposable gloves were worn to aseptically collect fresh rectal contents from the anus in 2-mL sterile Corning cryovials (Corning Incorporated, New York, USA) after cleaning, disinfection, and stabilization. The samples were labeled, quickly frozen in liquid nitrogen, kept in a -80 °C refrigerator.

DNA was extracted from the samples using the E.Z.N.A.® Soil DNA Kit following the manufacturer’s instructions and stored in a -80 ℃. NanoDrop2000 was used to detect DNA purity and concentration, and 1% agarose gel electrophoresis was performed to detect DNA integrity. The voltage was 5 V/cm, and the time was 20 min.

The extracted genomic DNA was submitted to a testing laboratory (Shanghai Majorbio Bio-pharm Technology Co., Ltd) for metagenomic sequencing. DNA fragments with an average size of approximately 400 bp were obtained using sonication. Paired-end sequencing was performed on an Illumina NovaSeq platform.

Additionally, we obtained metagenomic data from Qinghai (QH, 2 500 m above sea level) and Dezhou area (DZ, altitude ＜30 m) published in the previous article [17]. In total, data from 30 samples from six regions were used for the subsequent analysis.

### Data Processing

The software fastp [18] (https://github.com/OpenGene/fastp, version 0.20.0) was used to perform quality shearing on the adapter sequences at the 3′ and 5′ ends of the reads. The sheared length was < 50 bp; the average base quality value was < 20; the reads containing nitrogenous bases were removed to retain high-quality pair-end reads and single-end reads.

The optimized sequences were assembled using the splicing software MEGAHIT [19] (https://github.com/voutcn/megahit, version 1.1.2) based on the principle of succinct de Bruijn graphs. Contigs with a length of > 300 bp in the assembly results were screened as the final assembly results. Open Reading Frame (ORF) prediction of contigs in the splicing results was performed using MetaGene [20] (http://metagene.cb.k.u-tokyo.ac.jp/). Genes with nucleic acid lengths of ≥ 100 bp were selected and translated into amino acid (AA) sequences.

CD-HIT [21](http://www.bioinformatics.org/cd-hit/, version 4.6.1) was used to cluster the predicted gene sequences of all samples (parameters: 90% identity, 90% coverage), and the longest gene in each class was regarded as the representative sequence to construct a non-redundant (NR) gene set. Using the SOA Paligner [22] software (http://soap.genomics.org.cn/, version 2.21), the high-quality reads of each sample were separately aligned with the NR gene set (95% identity) and statistical gene abundance information in corresponding samples.

### Species and functional annotations

The AA sequences of the NR gene sets were aligned with the NR database using Diamond [23] with an expected e-value of 1e-5, and species annotations were obtained through the taxonomic information database corresponding to the NR database. The sum of the gene abundances corresponding to the species was used to calculate the abundance of the species.

Diamond [23] was used to compare the eggNOG (version 4.5.1), Kyoto Encyclopedia of Genes and Genomes (KEGG) (version 94.2)[24], and Carbohydrate-Active enZYme (CAZy) databases; the expected e-value was 1e-5 to obtain the corresponding function and calculate the abundance of functional categories.

### Redundancy analysis (RDA) correlation analysis of environmental factors

RDA was a constrained Principal Component Analysis, which can reflect samples and environmental factors on the same two-dimensional ranking graph, from which we intuitively observed the relationship between sample distribution and environmental factors. It comprised the following three steps: (1) The Bioenv function was used to determine the maximum Pearson correlation coefficient between the environmental factors and the distribution difference of species/function of the sample, and the subset of environmental factors was obtained by the maximum correlation coefficient. (2) RDA analysis was performed between the species/function distribution table of the sample and environmental factors or their subsets. (3) The significance of RDA analysis was determined by Permutest analysis, which is similar to the Analysis of Variance.

## Results

### Statistics

After filtering, a total of 2,341,055,166 reads and 353,195,288,355 bp were obtained. After the assembly, a total of 28,097,975 contigs were received with an average length of 21,921,556,352 bp and N50 of 26,337 bp. Finally, 198,223,248,57 open reading frames were generated. Afterward, analysis was performed using the NR, eggNOG, KEGG, and CAZymes databases.

### Compositional and differential analysis based on species abundance

**Fig. 2:**
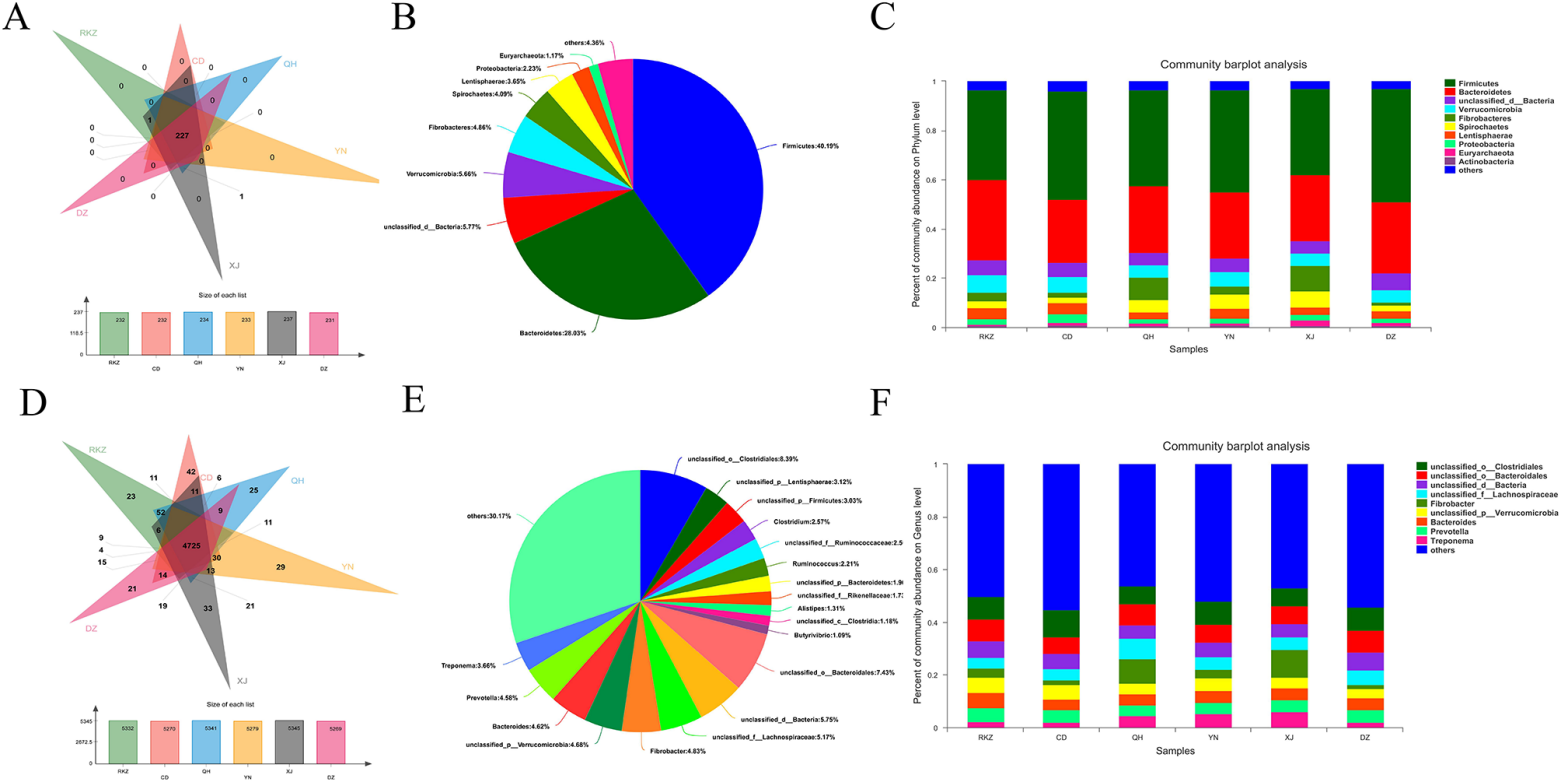
Species composition analysis at the phylum level **(A, B, and C)** and genus level **(D, E, and F)**. **(A and D)** Venn diagram. The numbers in the overlapping part represent the number of species shared by multiple groups, and the numbers in the non-overlapping part represent the number of species unique to the corresponding grouping. The figure below the Venn diagram is a column chart of the total number of species in each grouping. **(B and E)** The species distribution pie chart indicates the distribution of each species; different colors represent different species; the area of the pie chart represents the percentage of the total number of species to the total number of species. **(C and F)** Community column chart. The abscissa represents the sample name; the represents the proportion of species in the sample; the columns of different colors represent different species; the length of the column represents the proportion of the species

**Fig. 3:**
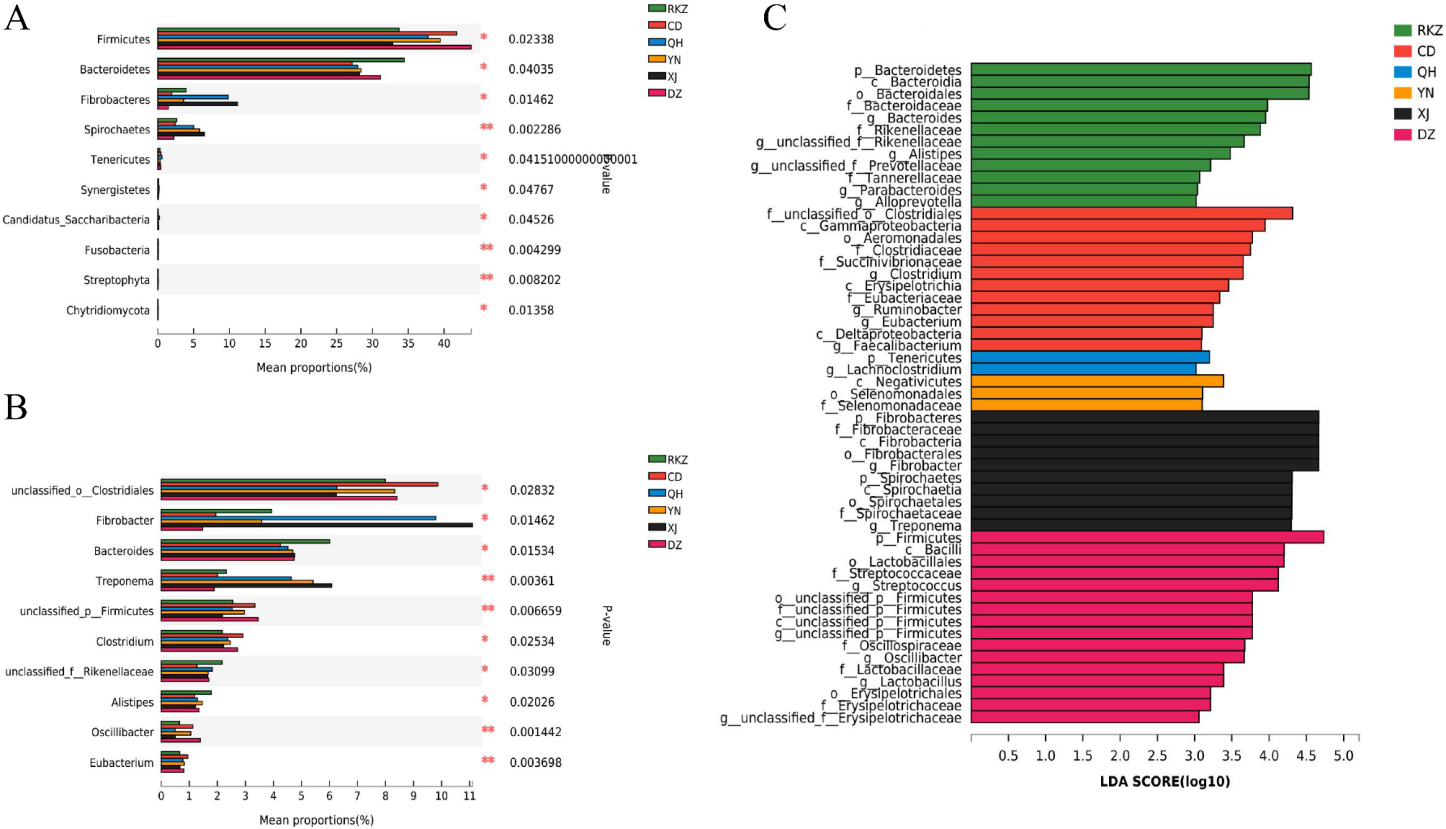
Comparison of multiple groups at the phylum level **(A)** and genus level **(B)**. The ordinate represents the species names at different taxonomic levels; the abscissa represents the percentage of the abundance of a certain species in the sample; different colors represent different groups. **(C)** Latent Dirichlet Allocation (LDA) diagram. The abscissa represents the LDA score; the greater the LDA score, the greater the influence of species abundance on the differential effect, and different colors represent different groups. *P < 0.05, ** *P < 0.01*, *** *P < 0.001*

At the phylum level, 227 common phyla were identified (Fig. 2A), and Firmicutes and Bacteroidetes were the dominant phyla in all intestinal sites (40.19% and 28.03% on average, respectively) (Fig. 2B). The dominant phyla were Bacteroidetes in RKZ and Firmicutes in other five donkey populations. Firmicutes accounted for the highest proportion in DZ; Bacteroidetes were the most abundant in RKZ. The abundance of Fibrobacteres rectal contents was significantly higher in XJ than that in DZ (*P < 0.05*). Spirochaetes had the highest abundance in XJ, which was significantly higher than that in RKZ (*P < 0.05*), CD (*P < 0.01*), and DZ (*P < 0.01*) (Fig. 3A).

At the genus level, 4,725 common genera were identified (Fig. 2D), and the dominant genera were *Clostridiales* and *unclassified_o_ Bacteroidales* (short for *Bacteroidales*) in all intestinal sites (8.39% and 7.43% on average, respectively) (Fig. 2E). The dominant genera were *Clostridiales* in RKZ, CD, YN and DZ; *Fibrobacter* in QH and XJ. The abundance of rectal contents of *Clostridiales* was the highest in CD, which was significantly higher than that in QH (*P < 0.05*) and XJ (*P < 0.05*). The abundance of *Fibrobacter*’s rectal contents was the highest in XJ, which was significantly higher than that in DZ (*P < 0.05*) (Fig. 3B).

It was worth noting that the proportions of Fibrobacteres in the XJ and QH regions and *Spirochaetes* in the QH, YN, and XJ regions were higher than those in other regions; *Lachnospiraceae* had the highest proportion in the QH region (Fig. 2C, F). As presented in the Latent Dirichlet Allocation (LDA) diagram in Fig. 3C, the key discriminators were *Bacteroidetes* in RKZ, *Clostridiales* in CD, *Tenericutes* in QH, *Negativicutes* in YN, *Fibrobacteres* in XJ, and *Firmicutes* in DZ. These results demonstrated significant differences in the microbiota composition among donkeys from six regions.

### Compositional and Differential Analysis based on functional abundance

**Fig. 4:**
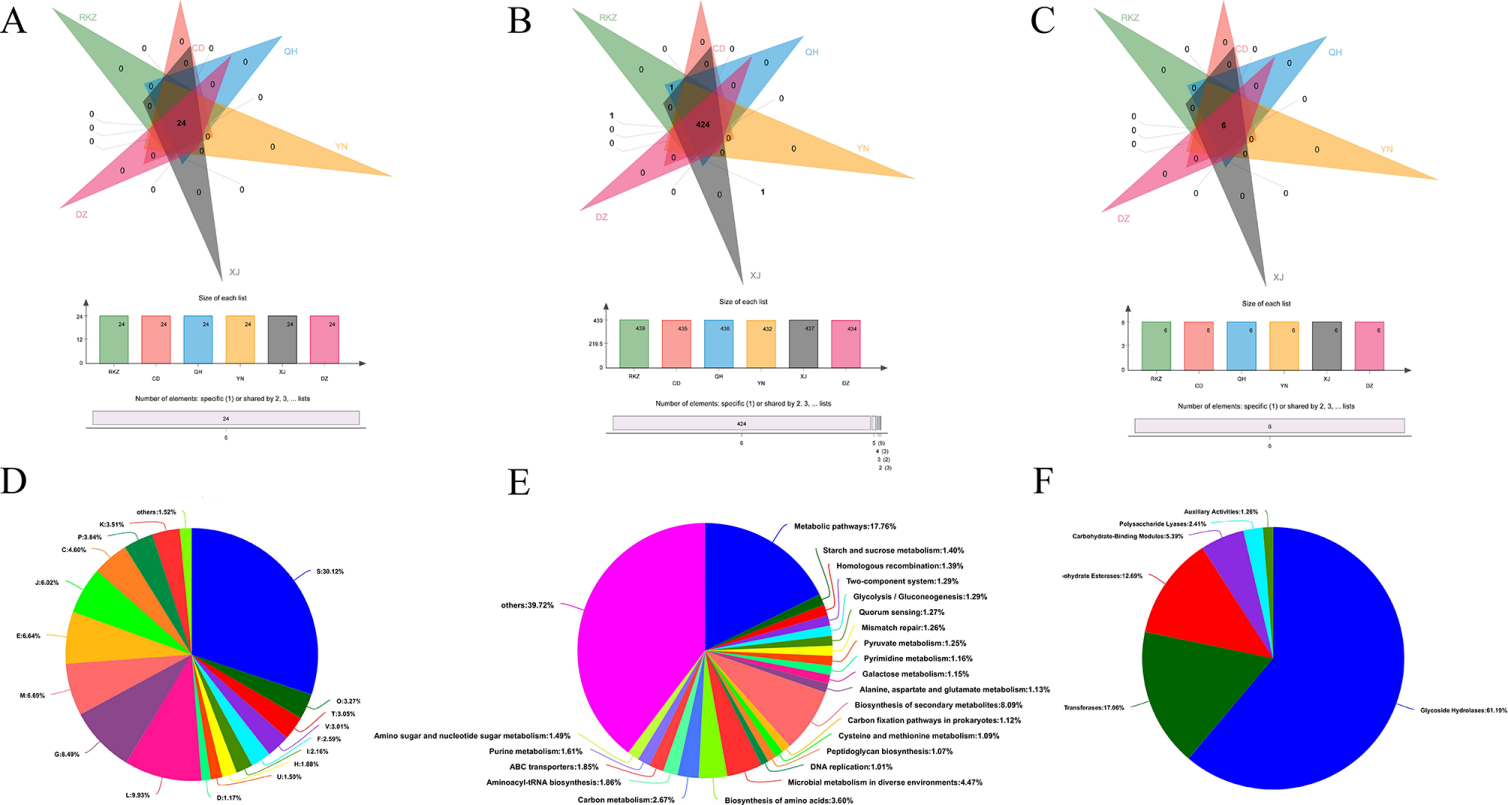
Venn diagrams of functional composition in clusters of orthologous genes (COG) **(A)**, Kyoto Encyclopedia of Genes and Genomes (KEGG) **(B)**, and Carbohydrate-Active enZYme (CAZy) **(C)**. Overlaps indicate features common to multiple sample groups; non-overlaps indicate features unique to sample groups, and numbers indicate the number of corresponding features. Pie charts of function distribution in COG **(D)**, KEGG **(E)**, and CAZy **(F)** represent the distribution of each function. Different colors represent different functions, and the area of the pie chart represents the percentage of the number of functions to the total number of functions

**Fig. 5:**
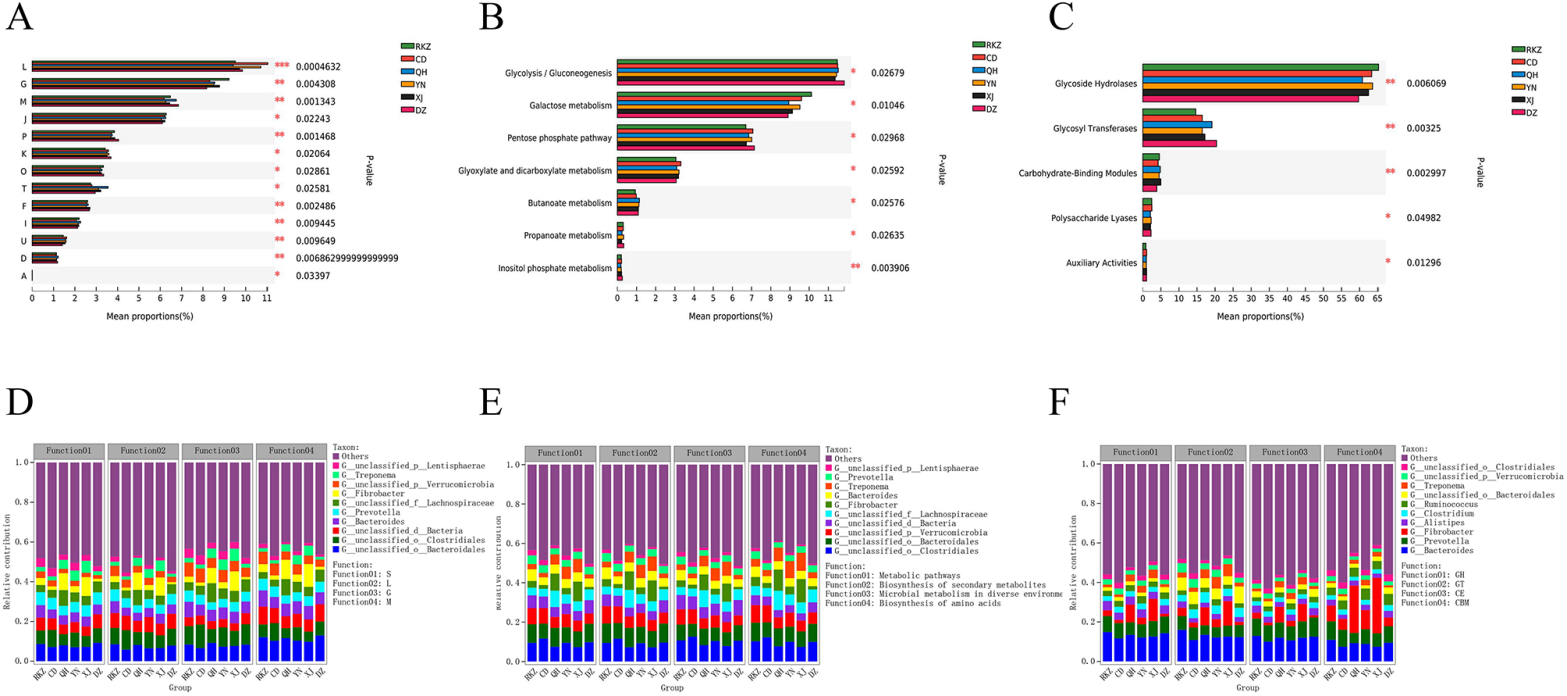
Column chart of functional difference analysis in clusters of orthologous genes (COG) **(A)**, Kyoto Encyclopedia of Genes and Genomes (KEGG) **(B)**, and Carbohydrate-Active enZYme (CAZy) **(C)**. The ordinate represents the name of species at different taxonomic levels; the abscissa represents the percentage value of the abundance of a certain species in the sample; different colors represent different groups. Species and functional contributions are indicated in COG **(D)**, KEGG **(E)**, and CAZy **(F)**: The abscissas in the figures represent the corresponding sample groups, and the ordinates represent the relative contributions. *P < 0.05, ** *P < 0.01*, *** *P < 0.001*

Among the clusters of orthologous genes (COG) functions, 24 functions were shared by the six groups (Fig. 4A). After removing the function unknown [S] (Function unknown, 30.12%), the top two with the highest abundance were replication, recombination, and repair [L] (9.93%) and carbohydrate transport and metabolism [G] (8.49%) (Fig. 4D). The functional abundance of [L] was highest in CD. The abundance of the [G] function was the highest in RKZ, and among this function, *Lachnospiraceae* had the highest contribution in the QH group. The functional abundance of cell wall/membrane/envelope biogenesis [M] was highest in DZ. Among [M] functions, *Bacteroidales* and *Bacteria*, *Clostridiales*, and *Fibrobacter* had high contributions in the DZ, CD, and XJ groups, respectively (Fig. 5A, D).

In the KEGG function, 424 functions were shared by the six groups (Fig. 4B), of which the top two with the highest abundance were metabolic pathways (17.76%) and biosynthesis of secondary metabolites (8.09%) (Fig. 4E). The functional abundance of glycolysis/gluconeogenesis was highest in DZ and significantly higher than that in XJ (*P < 0.05*); there were no significant differences in the remaining groups. The functional abundance of galactose metabolism was highest in RKZ, which was significantly higher than that of QH and DZ (*P < 0.01*) and XJ (*P < 0.05*). Pentose phosphate pathway abundance was highest in DZ, which was significantly higher than that in RKZ (*P < 0.05*) (Fig. 5B). Additionally, *Clostridiales* exhibited a high contribution to the function of microbial metabolism in diverse environments and CD groups. *Bacteroidales* presented a high contribution to the function of biosynthesis of secondary metabolites and DZ. *Verrucomicrobia* contributed more to the biosynthesis of secondary metabolites and RKZ. *Bacteria* had higher microbial metabolism in diverse environments and contributed more in DZ; *Lachnospiraceae* had a higher contribution in the biosynthesis of AA and QH; the contribution of *Fibrobacter* was significantly higher in the biosynthesis of AA and the XJ group than in other groups (Fig. 5E).

In the CAZy analysis, the six groups of gut microbiota covered six major enzymes and shared six functions (Fig. 4C), of which the first two with the highest abundance were glycoside hydrolase (GH) and glycosyl transferase (GT) (Fig. 4F).

At the class level, the functional abundance of GH was the highest in RKZ, which was significantly higher than that of QH (*P < 0.01*) and DZ (*P < 0.001*). *Bacteroides* contributed more to GH function and donkey’s gut microbes in the RKZ region. The functional abundance of GT was the highest in DZ, which was significantly higher than that in CD (*P < 0.05*), YN (*P < 0.05*), and RKZ (*P < 0.001*), and *Clostridium* had the highest contribution in GT function and donkey’s gut microbes in the QH region. The functional abundance of carbohydrate-binding modules (CBM) was significantly higher in XJ than that in DZ (*P < 0.01*) and QH than that in DZ (*P < 0.05*), respectively. The contribution of *Fibrobacter* in the CBM function and XJ group was significantly higher than that in other groups (Fig. 5 C, F).

**Fig. 6:**
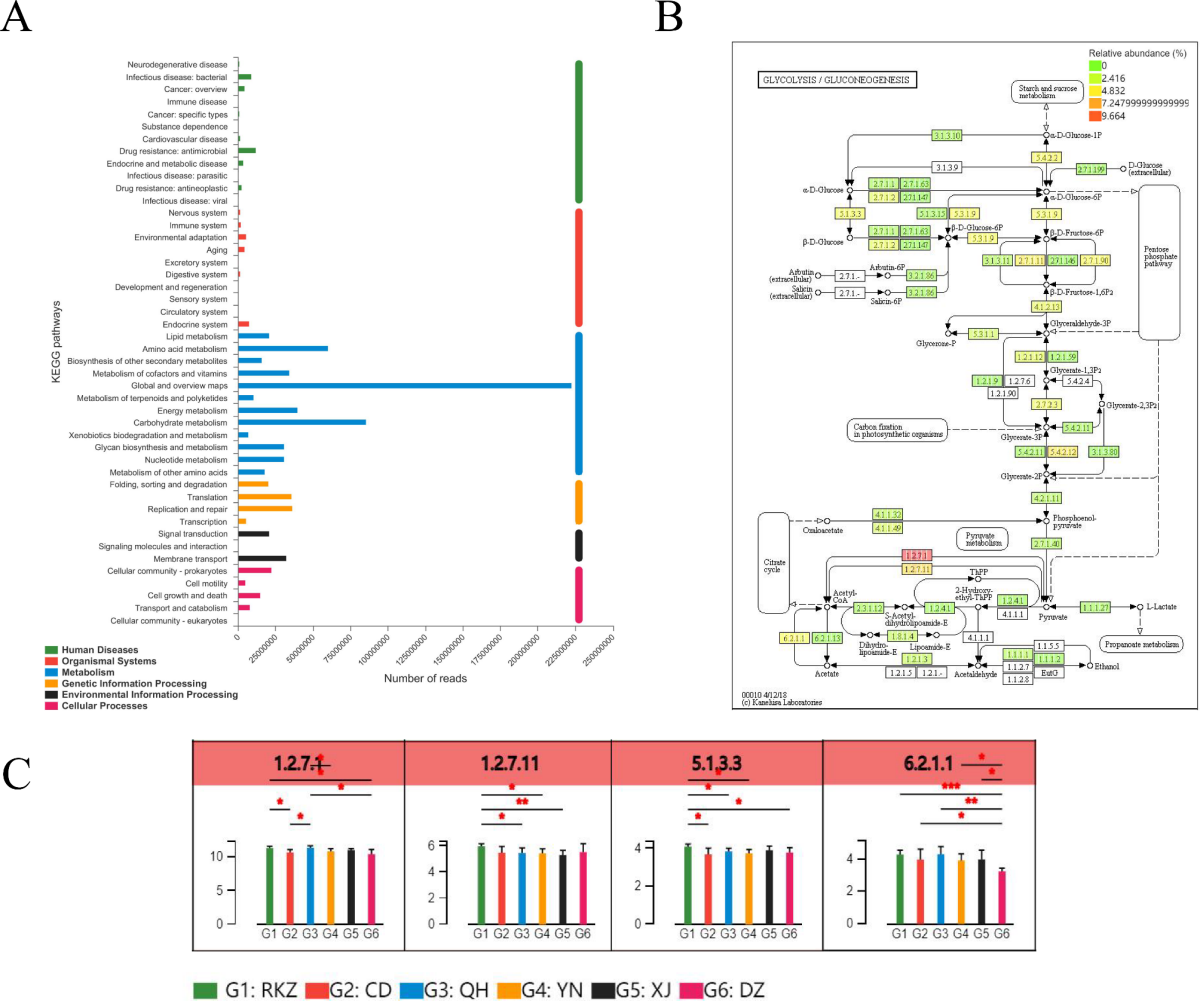
Differences between metabolite groups. **(A)** Pathway classification statistics histogram. The ordinate represents the function name of KEGG Pathway Level 2, and the abscissa represents its corresponding abundance value. The histogram is colored according to the Kyoto Encyclopedia of Genes and Genomes (KEGG) Pathway Level 1 to which KEGG Pathway Level 2 belongs. **(B)** Metabolic pathway difference test chart between groups. Each square in the box with a fill color in the figure represents one sample or a group of samples, and the intensity of the color represents the change in abundance of the enzyme in different samples or groups. **(C)** Histogram of differences in metabolic pathways between groups. In the legend, different colors correspond to different groups. *P < 0.05, ** *P < 0.01*, *** *P < 0.001*

Based on the KEGG annotation results, the differential detection and visual analysis of differentially abundant enzymes were performed for a certain pathway. At Level 2, metabolism had the highest functional enrichment, and the top three functional abundances were global and overview maps, carbohydrate metabolism, and AA metabolism (Fig. 6A). The most abundant carbohydrate pathway was glycolysis/gluconeogenesis (Fig. 6B), and the differences in their metabolic pathways are presented in Fig. 6B C. Among them, the abundance of pyruvate ferredoxin oxidoreductase (EC: 1.2.7.1, including four subunits, porA, porB, porG, and porD), in QH was the highest, which was higher than that in CD, YN, and DZ (*P < 0.05*); the abundance of pyruvate ferredoxin oxidoreductase in RKZ was higher than that in CD (*P* < 0.05) and DZ (*P* < 0.01). The abundance of 2-oxoglutarate/2-oxoacid ferredoxin oxidoreductase subunit alpha (EC: 1.2.7.11, including korA and korB subunits) in RKZ was the highest, which was higher than that in XJ (*P < 0.01*), QH, and YN (*P < 0.05*). The abundance of Aldose 1-epimerase (EC: 5.1.3.3, including galM subunits) in RKZ was the highest, which was higher than that in CD, QH, YN, and DZ (*P < 0.05*). The abundance of Acetyl-CoA synthetase (EC: 6.2.1.1, including two subunits of ACSS and AAE7) was highest in QH and the lowest in DZ, which was significantly lower than that in CD, YN, and XJ (*P < 0.05*), RKZ (*P < 0.001*), and QH (*P < 0.01*).

### Altitude Association Analysis based on species and functional abundance

**Fig. 7:**
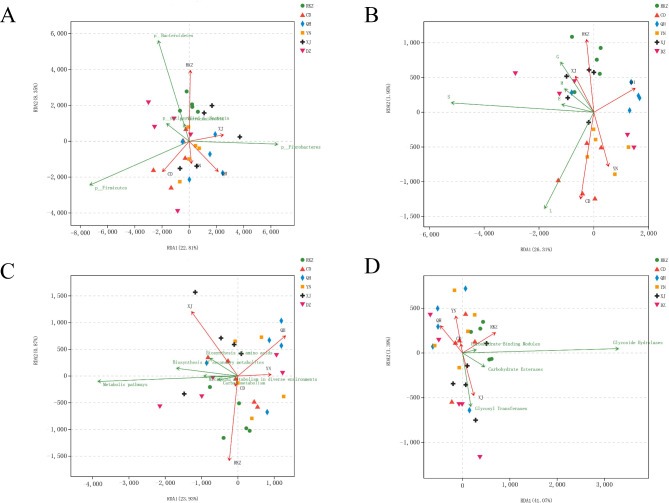
Association analysis of species **(A)**, clusters of orthologous genes (COG) **(B)**, Kyoto Encyclopedia of Genes and Genomes (KEGG) **(C)**, and Carbohydrate-Active enZYme (CAZy) **(D)** with altitude factors. The points in the figure represent samples; the different colors and shapes of the points represent different groups; the distance between the points represents the similarity and difference of functional composition between samples; the elevation factors/species are represented by arrows. The length of the line connecting the arrows represents the degree of correlation between the altitude factor/species and species distribution. The longer the line, the greater the correlation, and the shorter the line, the lower the correlation. The projected distance from the sample point to the quantitative altitude factor vector represents the degree to which the sample is affected by the altitude factor, i.e. the two samples draw a vertical line to the same environmental factor vector; the closer the projection line is, the more similar the influence of this altitude factor on the two samples is. The angle between the altitude factors represents the positive and negative correlation between the altitude factors (acute angle: positive correlation; obtuse angle: negative correlation; right angle: no correlation)

The top five species or functions in the samples from six different areas were examined for connection with altitude using RDA analysis. At the species level, Bacteroidetes was closest to RKZ (5 000 m) in the area with the highest altitude (Fig. 7A). In the COG function, the function of carbohydrate transport and metabolism [G] was the highest in the RKZ (5,000 m) group, and the maximum functional abundance of replication, recombination, and repair [L] was observed in the CD (3,500 m) (Fig. 7B). In the KEGG analysis, the function abundance of the metabolic pathway exhibited the maximum correlation with the RKZ (5,000 m) region (Fig. 7C). At the class level in the CAZy function, the RKZ (5,000 m) region had the highest correlation with the function of the GH (Fig. 7D).

## Discussion

### Analysis of gut microbiota composition based on species abundance

Firmicutes, followed by Bacteroidetes, was the most prevalent phylum at the phylum level in the six regions. The maximum abundance of Firmicutes, Bacteroidetes, and Fibrobacteres were observed in DZ donkeys, RKZ, and XJ, respectively. Previous studies have shown that Firmicutes and Bacteroidetes are the dominant microbial communities in the gut of horses [25]. Research by M Denise Dearing et al. showed that Firmicutes is the main microbial phylum that promotes the breakdown of fibers in the gastrointestinal tract of herbivores [26]. Cheryl Spence et al. discovered that Bacteroidetes is the main microbial phylum for carbohydrate metabolism in herbivores [27]. A study by Wu et al. demonstrated that Fibrobacteres microorganisms can facilitate the digestion of cellulose and hemicellulose in the diet [28]. This has also been supported by previous studies on the diversity of mammalian gut microbia [29, 30].

The host’s diet and mucosal secretions were mostly sources of polysaccharides in the gut. Most Firmicutes produce butyric acid, which is not absorbed by the intestine, and one of the final metabolites of polysaccharides is also butyric acid. Butyric acid was crucial for physiological activities such as providing energy and promoting the development of intestinal epithelial cells. Although ATP-binding transporters can be employed for carbohydrate conversion and transport, Firmicutes encode fewer carbohydrate-degrading enzymes [31]. *Bacteroides* can break down carbohydrates in food and provide about one-tenth of the energy to the host. It had been reported that *Bacteroides* can also resist the invasion of pathogens and enhance the body’s resistance by colonizing the intestinal mucosal epithelium [32]. About 20% of the genes in *Bacteroidetes* are used for the decomposition of sugars, which can efficiently degrade polysaccharides and produce a significant amount of short-chain fatty acids, was one of the most studied bacteria on polysaccharides [33].

At the genus level, the most abundant genus was *Clostridiales*, followed by *Bacteroidales*. *Clostridiales* and Bacteroidales had the highest abundance in CD and RKZ, respectively. *Clostridiales* is a cellulose-degrading bacteria capable of breaking down cellulose and providing fermentation substrates for acid-producing bacteria [34, 35]. *Clostridiales* species act as fiber fermenters to produce acetate and propionate from succinic acid produced by other bacteria [36, 37]. By improving the gastrointestinal flora, *Clostridiales* can stabilize the environment and intestinal pH, provide a favorable environment for the growth of gastrointestinal anaerobic flora, enhance the decomposition, digestion, absorption, and utilization of the gastrointestinal tract, improve the growth performance of animals, and enhance the body’s immune function. 16 S ribosomal RNA gene sequencing revealed that cocci bacteria and *Clostridium* were dominant in the rumen of Tibetan antelope, Tibetan wild donkey, and Tibetan sheep [38]. As ubiquitous hindgut symbionts in humans and other animals, many species of *Bacteroidetes* help digest various plant polysaccharides, including fibrous matter [39, 40]. In addition to their degradative activity, *Bacteroides* also play a crucial role in developing epithelial immunity and maintaining intestinal microecological balance [41]. Compared with that in the low-altitude wild mice, the proportion of *Bacteroides* in the intestinal tract of the plateau wild mice was significantly increased [42]. These results indicated that donkeys in the CD area had strong microbial metabolic function and those in the Shigatse region had strong glucose metabolism and utilization ability to adapt to the plateau environment.

### Analysis of gut microbiota composition based on functional abundance

The results of COG functional annotation revealed that replication, recombination, and repair [L], carbohydrate transport and metabolism [G], and cell wall/membrane/envelope biogenesis [M] had the highest functional abundance in CD, RKZ, and DZ, respectively. The main pathways annotated in the KEGG database include metabolic pathways and biosynthesis of secondary metabolites, among which glycolysis/gluconeogenesis had the highest functional abundance.

GH and GT were the most abundant among the CAZy functional annotations. GH accounted for the highest proportion in RKZ, and according to the LDA discrimination results, the GH2 function had a significant effect on RKZ. The GH2 family contains a large number of glycosidases that cleave oligosaccharides and non-reducing carbohydrates in the side chains of hemicellulose and pectin. Furthermore, in the KEGG functional annotation, the top two including glycan degradation and galactose metabolism was located in RKZ, indicating that RKZ samples exhibited a strong sugar metabolism. Among various carbohydrate enzymes, GHs, including glycosidases and transglycosidases, which were the most abundant and diverse group of enzymes responsible for the hydrolysis or transglycosylation of glycosidic bonds, accounted for half of the enzymes in the CAZymes database [43], and these can break glycosidic bonds of polysaccharides in plants [44]. Studies have confirmed that there are 72 different GHs families in buffalo and 78 different GHs in the rumen of cattle, which are responsible for the hydrolysis of various cell wall components [45]. In the CAZy database annotations, GT accounted for the highest proportion in DZ. Previous studies have reported that GT was responsible for catalyzing the cleavage of glycosidic bonds, accounting for 19–24% of the total CAZyme, and was the second most abundant enzyme in the CAZy family [46]. Carbohydrate-binding molecules exhibit no enzymatic activity but can bind GHs, CEs, and AAs to polysaccharides, thus, increasing their activity [47]. CEs can remove the ester group modification present in polysaccharides, thus, promoting the effect of GHs on complex polysaccharides. This may be associated with the fact that donkeys in RKZ have a unique intestinal flora to adapt to the low temperature, hypoxia, and high altitude of the plateau. Meanwhile, the correlation analysis based on environmental factors (Fig. 7) revealed that the altitude of the RKZ area had the highest correlation with the donkey’s intestinal microbes. It exhibited that the composition and function of intestinal flora will be affected by the severe environment conditions such as low temperature and hypoxia in high altitude area, however, the specific mechanism of action still needs additional research and data support.

The glycolysis/gluconeogenesis pathways for carbohydrates were enriched in KEGG, with pyruvate ferredoxin oxidoreductase being the most abundant in QH. 2-oxoglutarate/2-oxoacid ferredoxin oxidoreductase subunit alpha and aldose 1-epimerase were most abundant in RKZ, and Acetyl-CoA synthetase is most abundant in QH. All tested archaea contained pyruvate ferredoxin oxidoreductase, which is involved in either catabolism or anabolism. Archaea are single-celled microorganisms that do not have a nucleus and any other membrane-bound organelles, and in the metabolic process, there are many special coenzymes, most of which are strictly anaerobic, facultative anaerobic, and obligate aerobic [48]. Thus, pyruvate ferredoxin oxidoreductase represents the only mechanism for pyruvate acetyl-CoA conversion in the archaeal domain [49]. Aldose 1-epimerase or mutarotase was a key enzyme in carbohydrate metabolism that catalyzes the interaction between α-D-glucose and β-D-glucose and their mutual transformation. This enzyme is necessary for normal lactose metabolism in *E. coli.* Strains, in which the gene that had been deleted grew more slowly on a medium containing any sugar. Mutarotases have been reported to convert various sugars, including D-glucose, D-fucose, D-quinose, L-arabinose, and D-xylose. A crucial metabolite in the metabolism of carbon and energy is acetyl-CoA. In mammalian cells, there are many ways for carbohydrates to be converted into various biomolecules. One of these included the breakdown of carbohydrates into acetyl-CoA, which was subsequently employed as a precursor for anabolism. This was a crucial technique for transforming sugars into other biomolecules. The three main pathways of mitochondrial acetyl-CoA synthesis include the oxidative decarboxylation of glycolysis product pyruvate, fatty acid β-oxidation, and branched-chain AA decomposition. Mitochondrial acetyl-CoA normally progresses to the tricarboxylic acid cycle for further metabolism [50]. This indicated that donkeys in high-altitude regions had relatively strong carbohydrate metabolism and sugar conversion, which can fulfil the consumption of basal metabolism in the high-altitude environment and improve hypoxia endurance to help them adapt.

## Conclusion

In this study, we analyzed the gut flora of six donkey populations by metagenomic high-throughput sequencing. We observed that Firmicutes and Bacteroidetes were the dominant phyla, and the dominant genera were *Clostridiales* and *Bacteroidales*. Additionally, the donkey flora structure and function had significant differences in different areas. We hope that this study will serve as a specific reference for understanding the gut microbiome structure of donkeys in China and close a research gap in donkey gut microbiome research.

## Data Availability

The datasets generated in this study have been uploaded to National Center for Biotechnology Information repositories and will be made available after the article is received. Shigatse, Changdu, Yunnan, and Xinjiang areas of donkey gut microbe data repository/repository name and registration number can be found on the following site: https://www.ncbi.nlm.nih.gov/, BioProject ID: PRJNA894235. Qinghai and Dezhou areas of donkey gut microbe data repository/repository name and registration number can be found on the following site: https://www.ncbi.nlm.nih.gov/, BioProject ID: PRJNA843204[17].
